# Extracellular Vesicles Derived from Human Umbilical Cord Mesenchymal Stem Cells Alleviated the Inflammatory Response in Mice Infected with the Influenza Virus A (H1N1)

**DOI:** 10.3390/ijms26188839

**Published:** 2025-09-11

**Authors:** Hui Xiao, Xiao Yu, Yiding Dong, Shilong Bao, Xiaoting Meng, Jia Zhao, Zhiyong Dong

**Affiliations:** 1 The Key Laboratory of Pathobiology, Ministry of Education, College of Basic Medical Sciences, Jilin University, Changchun 130021, China; 2 Department of Histology & Embryology, College of Basic Medical Sciences, Jilin University, Changchun 130021, China; 3 Department of Forensic Medicine, College of Basic Medical Sciences, Jilin University, Changchun 130021, China

**Keywords:** extracellular vesicles derived from human umbilical cord mesenchymal stem cells (hUCMSC-EVs), influenza A virus (H1N1), lung injury, inflammatory response, cytokines

## Abstract

Influenza A virus (H1N1) infection poses a significant global public health challenge and imposes a substantial economic burden. Numerous studies have shown that excessive immune activation and dysregulated inflammatory responses following influenza virus infection are the primary causes of disease progression and mortality. Extracellular vesicles derived from mesenchymal stem cells (MSC-EVs) exhibit potent anti-inflammatory effects. Therefore, this study aims to investigate the effects of extracellular vesicles derived from human umbilical cord mesenchymal stem cells (hUCMSC-EVs) on pulmonary inflammatory responses in mice infected with the influenza A virus (H1N1). The study first established a mouse influenza virus infection model by intranasal inoculation of the influenza A virus (H1N1), followed by treatment with hUCMSC-EVs (70 μg) administered via tail vein injection for four consecutive days. The results showed that compared with the H1N1 group, after treatment with hUCMSC-EVs, pulmonary edema was reduced, inflammatory cell infiltration in the lungs was significantly decreased, and the expression levels of tumor necrosis factor-α (TNF-α), interleukin-1β (IL-1β), and interleukin-6 (IL-6) proteins in serum and lung tissue were significantly reduced. Therefore, this study suggests that the protective effect of hUCMSC-EVs against lung damage caused by influenza A virus (H1N1) infection may be related to the reduction in inflammatory cytokine levels of TNF-α, IL-1β, and IL-6, thereby alleviating pulmonary inflammation.

## 1. Introduction

According to the World Health Organization (WHO), seasonal influenza epidemics result in approximately 3–5 million severe cases and 300,000–650,000 deaths annually, posing a major global public health challenge and imposing a significant economic burden [1]. Influenza viruses, the causative agents of influenza, are classified into four types (A, B, C, and D). Among these, influenza A virus (IAV) is the most clinically prevalent due to its high infectivity and propensity for mutation [2]. Currently, the main treatments for influenza viruses include vaccines and antiviral drugs. However, influenza viruses are prone to mutation and have many subtypes, which hinders the development and application of vaccines [3,4]. In addition, studies have shown that influenza viruses are gradually developing resistance to antiviral drugs such as M2 channel inhibitors (e.g., amantadine and rimantadine) and neuraminidase inhibitors (e.g., oseltamivir and zanamivir), and these drugs also have certain toxic side effects [5,6]. Therefore, there is an urgent need for research and development of effective and safe drugs for the treatment of influenza virus infections.

Studies have shown that excessive immune activation and dysregulated inflammatory responses following influenza virus infection are key drivers of disease progression and mortality [7]. Consequently, modulating hyperactive immune and inflammatory responses is critical in influenza treatment.

Mesenchymal stem cells (MSCs) are multipotent stromal cells with self-renewal capacity, multilineage differentiation potential, as well as important functions such as trophic, homing/migratory, and immunomodulatory functions, making them promising candidates for treating inflammatory diseases [8,9,10,11]. Emerging evidence suggests that MSCs primarily exert anti-inflammatory and immunomodulatory functions through paracrine pathways, with extracellular vesicles (EVs) being the most intriguing component [12]. EVs are small membrane vesicles (50 to 200 nm) released by almost all cell types, which mediate intercellular communication by transferring part of the membrane and cytoplasmic proteins, lipids, and nucleic acids [12,13,14]. Notably, EVs derived from MSCs (MSC-EVs) have demonstrated anti-inflammatory effects in inflammatory lung disorders such as chronic obstructive pulmonary disease (COPD) and asthma [14,15,16,17].

However, it remains unclear whether EVs derived from human umbilical cord MSCs (hUCMSC-EVs) can mitigate influenza A virus (H1N1)-induced lung inflammation. To address this gap, our study aimed to investigate the therapeutic potential of hUCMSC-EVs in a murine model of influenza A virus (H1N1) infection. We established an in vivo mouse model of influenza A virus (H1N1) infection and administered hUCMSC-EVs (70 μg) via tail vein injection continuously for four days starting 24 h after infection. At seven days post-infection (dpi), we evaluated body weight, core temperature, clinical scores, pulmonary index, histopathological changes, and inflammatory cytokine levels in both serum and lung tissue. Our findings revealed that hUCMSC-EVs treatment significantly attenuated lung inflammation and injury, potentially through the downregulation of pro-inflammatory cytokines, including tumor necrosis factor-α (TNF-α), interleukin-1β (IL-1β), and interleukin-6 (IL-6).

## 2. Results

### 2.1. Characterization of hUCMSCs and hUCMSC-EVs

**Figure 1:**
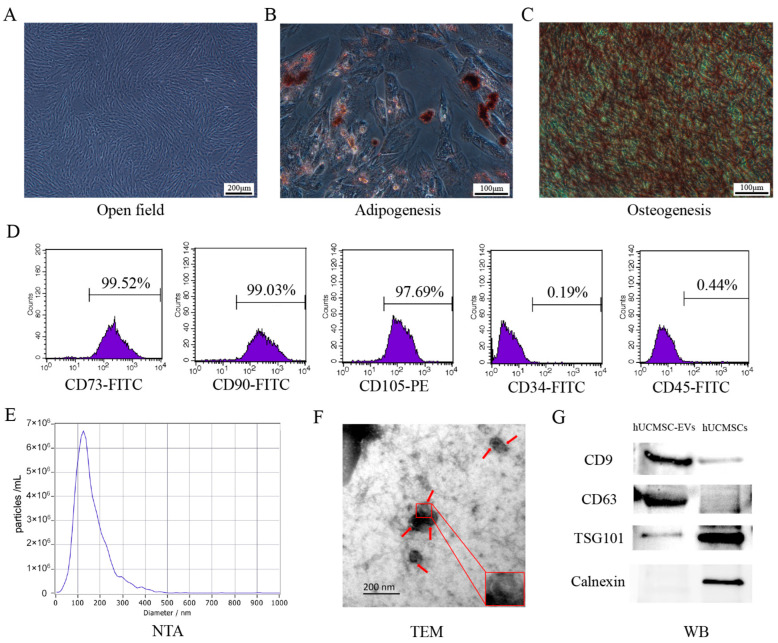
Characterization of human umbilical cord mesenchymal stem cells (hUCMSCs) and extracellular vesicles derived from hUCMSCs (hUCMSC-EVs). (**A**) hUCMSCs passed to the 3rd generation grew adherently to the wall and were in good condition, with the morphology of elongated pike/pike shape and the overall distribution of vortex (Scale bar = 200 μm). (**B**) Oil red O staining of hUCMSCs after lipogenic induction, visible with obvious red lipid droplets (Scale bar = 100 μm). (**C**) Alizarin red staining of hUCMSCs after osteogenic induction, visible with obvious red calcium nodules (Scale bar = 100 μm). (**D**) Identification of hUCMSCs surface markers by flow cytometry. (**E**) ZETAVIEW particle potential titration and particle size analyzer for hUCMSC-EVs particle size detection. (**F**) Transmission electron microscopy to observe hUCMSC-EVs morphology (scale bar = 200 nm), the red arrow indicates the hUCMSC-EVs. (**G**) Western blotting to identify the expression of hUCMSC-EVs marker protein.

hUCMSCs exhibited adherent growth with a spindle-shaped morphology and a characteristic swirling distribution (Figure 1A). The multidirectional differentiation potential of hUCMSCs has been demonstrated through adipogenic differentiation and osteogenic differentiation, which were confirmed through oil red O staining and alizarin red staining, respectively. The results showed the formation of obvious red lipid droplets and red calcium nodule (Figure 1B,C). Flow cytometry revealed high expression of MSC markers (CD73: 99.52%; CD90: 99.03%; CD105: 97.69%) and negligible expression of hematopoietic markers (CD34: 0.19%; CD45: 0.44%) (Figure 1D), confirming MSC identity.

To characterize hUCMSC-EVs, we first performed particle size analysis using nanoparticle tracking analysis. The results showed that the average size of the detected EVs was 158.8 nm, with a concentration of 8.3 × 10^10^ particles/mL, and the main peak was 123.6 nm, accounting for 99.4% of the total (Figure 1E). Then, the morphology of the isolated hUCMSC-EVs was observed by transmission electron microscopy (TEM), and the results indicated that they presented a typical dish-like shape and had intact cell membrane structures (Figure 1F). Finally, the expression of hUCMSC-EVs marker proteins was identified by using Western blotting. The results showed that hUCMSC-EVs expressed the positive markers CD9, CD63, and TSG101, while the negative marker Calnexin was not expressed (Figure 1G). Figure S1 displays SDS-PAGE gels treated with Coomassie Brilliant Blue staining and silver staining. These data validate the successful separation of hUCMSC-EVs.

### 2.2. hUCMSC-EVs Attenuated Clinical Symptoms in Influenza A Virus (H1N1)-Infected Mice

**Figure 2:**
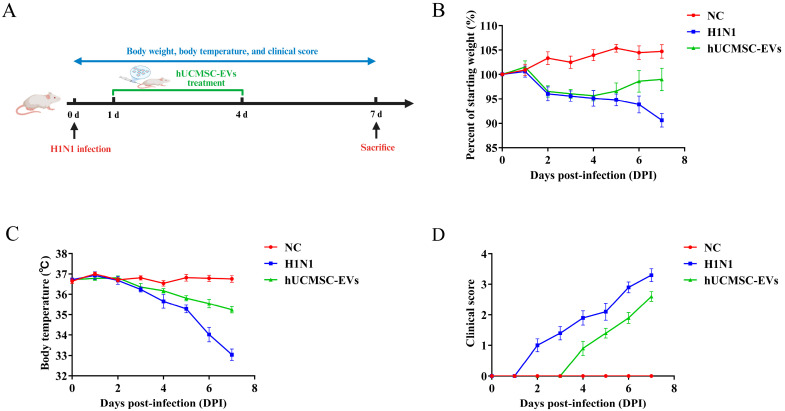
Extracellular vesicles derived from human umbilical cord mesenchymal stem cells (hUCMSC-EVs) have a significant protective effect on the survival of influenza virus A (H1N1)-infected mice. (**A**) Schematic overview of the animal experimental procedures. (**B**) The changes in body weight of the 3 groups of mice (NC group, H1N1 group, hUCMSC-EVs group) were observed and recorded for 7 consecutive days. (**C**) The changes in body temperature of the 3 groups of mice (NC group, H1N1 group, hUCMSC-EVs group) were observed and recorded for 7 consecutive days. (**D**) The clinical scores of mice in the 3 groups (NC group, H1N1 group, hUCMSC-EVs group) were observed and recorded for 7 consecutive days.

The body weight, body temperature, and clinical scores of three groups of mice (normal control (NC) group (uninfected and untreated), H1N1-infected (H1N1) group (infected and untreated), and hUCMSC-EVs-treated (hUCMSC-EVs) group (infected and treated)) were observed and recorded for seven consecutive days to determine the protective effect of hUCMSC-EVs on influenza virus A (H1N1)-infected mice. Mice were monitored for seven days post-infection (Figure 2A). The performance of mice in the hUCMSC-EVs group reflected a favorable protective effect throughout the entire study observation period. On the seventh day, the body weight of mice in the NC group was recorded as 19.58 ± 0.57 g, that of the H1N1 group was 16.91 ± 0.89 g, and that of the hUCMSC-EVs group was 18.43 ± 1.31 g. hUCMSC-EVs treatment partially restored weight (*p* < 0.01) (Figure 2B). Meanwhile, mice in the H1N1 group experienced hypothermia (H1N1 group: 33.04 ± 0.87 °C vs. NC: 36.75 ± 0.54 °C (*p* < 0.001)), and hUCMSC-EVs group improved temperature (35.24 ± 0.51 °C) (Figure 2C). Moreover, influenza virus A (H1N1)-infected mice showed lethargy, ruffled fur, and labored breathing by Day 1. hUCMSC-EVs treatment delayed onset and reduced severity of symptoms (Figure 2D).

### 2.3. hUCMSC-EVs Reduced Lung Pathology

**Figure 3:**
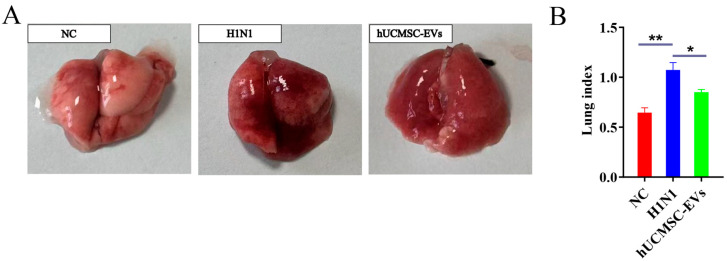
Gross anatomical observation of lung tissue and lung index test in each group of mice. (**A**) Gross anatomical observation of lung tissue of mice in each group. (**B**) Lung index test of mice in each group. Statistical significance was calculated from six independent experiments using two-tailed Student’s *t*-tests or one-way analysis of variance (ANOVA). * *p* < 0.05; ** *p* < 0.01.

On the seventh day after infection with the influenza A virus (H1N1), the gross anatomical results showed that the lung tissue of the NC group mice was pink, without dark red color. The lung tissue of the H1N1 group mice showed dark red color in one or more areas of the lung or even the entire lung, accompanied by edema. The gross autopsy of mice in the hUCMSC-EVs group revealed that compared with the virus infected group, the lung lesions were significantly reduced and the color was lighter, and the edema was also alleviated compared with those of the H1N1 group (Figure 3A).

Subsequently, the lung tissue of the mice was weighed, and the lung index of the mice was calculated. The results showed that the lung index was 0.65 ± 0.1 in the NC group, 1.07 ± 0.15 in the H1N1 group, and 0.85 ± 0.03 in the hUCMSC-EVs group. Although the lung index of the mice treated with hUCMSC-EVs group was increased compared with the NC group, it was significantly lower than that of the H1N1 group (Figure 3B).

### 2.4. hUCMSC-EVs Improved Lung Histopathology

**Figure 4:**
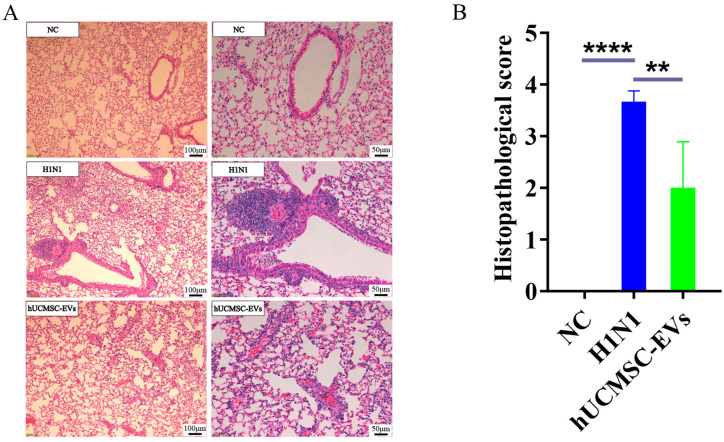
Extracellular vesicles derived from human umbilical cord mesenchymal stem cells (hUCMSC-EVs) significantly improved lung histopathological status of influenza A virus (H1N1)-infected mice. (**A**) H&E staining of lung tissue of mice in each group (scale bar = 100 μm, scale bar = 50 μm). (**B**) Statistics of lung histopathological scores of mice in each group. Statistical significance was calculated from four independent experiments using two-tailed Student’s *t*-tests or one-way analysis of variance (ANOVA). ** *p* < 0.01, **** *p* < 0.0001.

On the seventh day after infection with the influenza A virus (H1N1), the pathological changes in lung tissue were observed and evaluated through hematoxylin–eosin (H&E) staining. NC group: Normal alveoli and vasculature. H1N1 group: Unclear structure of alveolar tissue; alveolar wall thickening, fusion, and dilatation; interstitial edema; inflammatory infiltration; hemorrhage. hUCMSC-EVs group: Improved alveolar fusion and dilatation; reduced edema; minimal fibrosis; fewer inflammatory cells (Figure 4A). Histopathological scores were significantly lower in hUCMSC-EVs treated mice (Figure 4B).

### 2.5. hUCMSC-EVs Suppressed Systemic Inflammation

**Figure 5:**
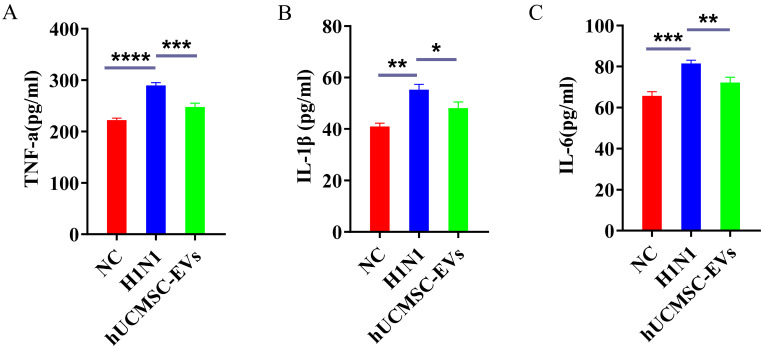
Extracellular vesicles derived from human umbilical cord mesenchymal stem cells (hUCMSC-EVs) significantly reduced inflammatory factors in serum of influenza A virus (H1N1)-infected mice. Comparison of serum levels of pro-inflammatory cytokine (**A**) tumor necrosis factor-α (TNF-α), (**B**) interleukin-1β (IL-1β), and (**C**) interleukin-6 (IL-6) in mice in each group (pg/mL). Statistical significance was calculated from five independent experiments using two-tailed Student’s *t*-tests or one-way analysis of variance (ANOVA). * *p* < 0.05, ** *p* < 0.01, *** *p* < 0.001, **** *p* < 0.0001.

Compared with the NC group, the serum levels of TNF-α, IL-1β, and IL-6 were significantly elevated in the H1N1 group of mice. hUCMSC-EVs reduced all three cytokines (Figure 5A–C).

### 2.6. hUCMSC-EVs Mitigated Lung Inflammation

**Figure 6:**
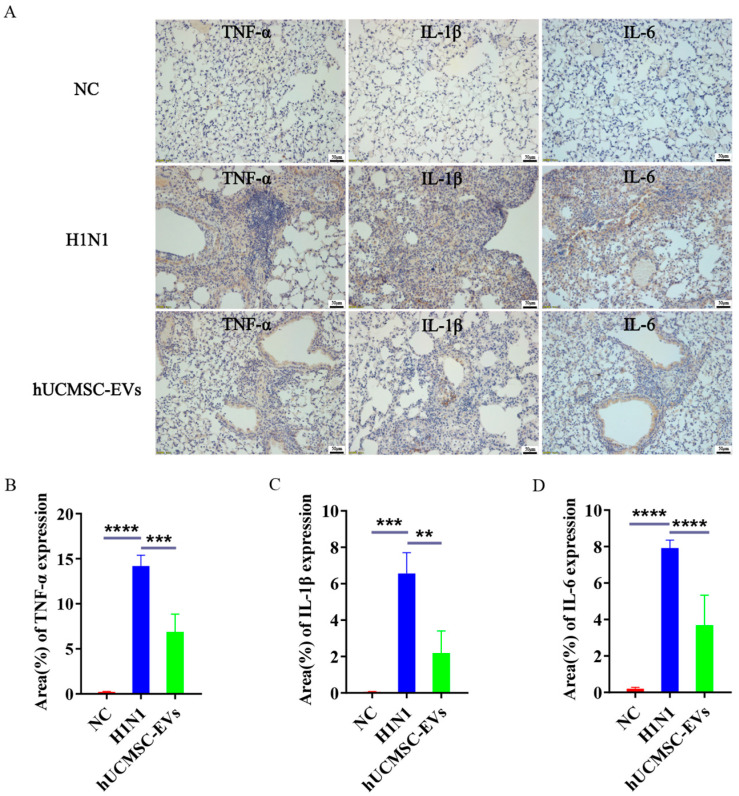
Extracellular vesicles derived from human umbilical cord mesenchymal stem cells (hUCMSC-EVs) significantly ameliorated lung inflammation in influenza A virus (H1N1)-infected mice. (**A**) Immunohistochemistry was performed to detect the expression of pro-inflammatory cytokines tumor necrosis factor-α (TNF-α), interleukin-1β (IL-1β), and interleukin-6 (IL-6) in the lung tissues of mice in each group. Image J analysis of the positive expression statistics of pro-inflammatory cytokines (**B**) TNF-α, (**C**) IL-1β, and (**D**) IL-6 in the lung tissues of mice in each group. Statistical significance was calculated from five independent experiments using two-tailed Student’s *t*-tests or one-way analysis of variance (ANOVA). ** *p* < 0.01, *** *p* < 0.001, **** *p* < 0.0001.

Compared with the NC group, the contents of TNF-α, IL-1β, and IL-6 in the lung tissues of H1N1 group mice were significantly increased, indicating that the lung tissue inflammation in mice infected with influenza A virus (H1N1) was more severe. Compared with the H1N1 group, the levels of TNF-α, IL-1β, and IL-6 in the lung tissues of the hUCMSC-EVs group mice were significantly decreased (Figure 6A–D), suggesting that after hUCMSC-EVs treatment, the levels of inflammatory factors TNF-α, IL-1β, and IL-6 in the lung tissues of mice were significantly reduced, and the lung inflammation was improved.

## 3. Discussion

Influenza A, a highly contagious respiratory disease, has caused numerous global outbreaks, posing a significant threat to public health. Although several antiviral drugs have been developed, their clinical utility is limited by adverse effects and the emergence of drug resistance [5,18]. Consequently, there is an urgent need for safer therapeutic alternatives. It has been found that after influenza virus infection, the body is over-immunologically activated and releases large amounts of pro-inflammatory cytokines (e.g., TNF-α, interferon-α (IFN-α), and IL-6), causing a cytokine storm that leads to inflammation in the lungs [19]. Thus, modulating this inflammatory cascade is critical for improving outcomes.

Currently, most treatments for influenza viruses focus on natural products and natural medicines. However, many natural products have issues such as low solubility, poor stability, and low bioavailability. While natural products can be combined with nanoparticle technology to address these issues, most are still in the preclinical research stage and require further clinical studies [20]. Studies have shown that umbilical cord MSCs (UCMSCs) can alleviate lung damage caused by influenza A virus (H1N1) infection [21]. However, previous studies have indicated that MSCs are susceptible to IAV infection, leading to increased cell death and potentially affecting their therapeutic efficacy [22].

MSC-EVs have emerged as promising candidates due to their cargo of bioactive molecules (e.g., mRNAs, microRNAs, cytokines, chemokines, immunomodulatory factors, etc.), which regulate immune cell function and mitigate inflammation [10]. Unlike whole MSCs, MSC-EVs offer advantages as a cell-free therapy, including reduced risks of allograft rejection, tumorigenicity, and embolism [23,24]. Preclinical studies support their efficacy: for instance, adipose-derived MSC-derived EVs (ADMSC-EVs) alleviated sepsis-induced lung injury by suppressing the inflammatory response and promoting transforming growth factor-β (TGF-β) secretion in macrophages [25]. Clinically, nebulized MSC-EVs improved outcomes in COVID-19 pneumonia without adverse reactions, shortening hospital stays [26]. Furthermore, hUCMSC-EVs ameliorated severe steroid-resistant asthma (SSRA) by modulating macrophage polarization via the TRAF1/NF-κB/PI3K/AKT axis [15], highlighting their broad anti-inflammatory potential.

Therefore, in this study, we selected hUCMSC-EVs for the treatment of influenza A virus (H1N1) infection. To evaluate the efficacy of hUCMSC-EVs, we established a murine model via intranasal viral inoculation and administered hUCMSC-EVs intravenously for 4 days. Key findings include the following: (1) Clinical improvement: hUCMSC-EVs attenuated weight loss, hypothermia, and clinical symptom severity (Figure 2). (2) Lung pathology: hUCMSC-EVs treatment reduced lung indices, edema, and hemorrhage (Figure 3), with histopathology confirming decreased inflammatory infiltration, alveolar wall thickening, and tissue disruption (Figure 4). In this study, the lung index (the ratio of lung weight to body weight) [27,28,29] was used as the primary indicator to quantify the severity of pulmonary edema, which is a typical feature in our model. Therefore, the significantly reduced lung index observed in the animals treated with hUCMSC-EVs provided direct evidence for the effective alleviation of the core pathological process of edema formation by this treatment. Histopathological analysis revealed a significant reduction in the thickening of alveolar walls and infiltration of inflammatory cells, confirming that the lower index reflects a true tissue protective effect. Furthermore, the reduction in lung weight was often consistent with the decrease in pro-inflammatory mediators (such as TNF-α, IL-1β, IL-6), indicating a broader anti-inflammatory effect. In summary, the observed reduction in lung index is a statistically significant and biologically relevant finding, consistent with previous results obtained using Lycium barbarum glycopeptide (LbGp) [30] and Geniposide [27] to treat influenza virus-induced reductions in lung index. (3) Cytokine modulation: hUCMSC-EVs significantly lowered TNF-α, IL-1β, and IL-6 levels in serum and lung tissue (Figure 5 and Figure 6), aligning with evidence that these cytokines drive lethal pneumonitis in influenza.

Previous studies have shown that ADMSC-EVs can improve pathological damage of lung tissue in mice caused by influenza A virus (H1N1), alleviating pulmonary edema, hemorrhage, and inflammatory cell infiltration. They can also significantly reduce the expression of pro-inflammatory cytokines such as IL-1β, IL-6, TNF-α, interferon-γ (IFN-γ), and interleukin-10 (IL-10), indicating that ADMSC-EVs have beneficial effects in influenza A virus (H1N1)-induced acute lung injury (ALI) [31]. Our results are consistent with those of previous studies.

However, EVs from different MSC sources have differences in functional molecules and targeting mechanisms. In lipopolysaccharide (LPS)-induced ALI, hUCMSC-EVs alleviate inflammation by inhibiting macrophage pyroptosis, which may be achieved by transferring pyroptosis-targeting miRNAs and immunomodulatory proteins through hUCMSC-EVs [32], while ADMSC-EVs mainly protect pulmonary endothelial cells from acute injury through the PI3K/Akt pathway [33]. In sepsis-induced ALI, ADMSC-EVs show more significant protective effects compared to EVs from bone marrow and umbilical cord sources [34]. However, there are no studies yet that have applied EVs from different sources of MSCs to lung injury caused by influenza A virus (H1N1) infection.

In this study, we selected hUCMSCs over other sources of MSCs due to their advantages of non-invasive collection, ease of acquisition, rapid proliferation, excellent immunomodulatory capacity, and anti-inflammatory effects, as well as the absence of ethical issues [35,36,37].

Additionally, the route of administration is also of great significance. In a mouse model of LPS-induced ALI, intravenous administration was more effective than intranasal or nebulized administration in reducing lung injury at equivalent doses, which may be related to the lung-targeting effect of intravenous administration [38]. In the lung injury model induced by swine influenza virus, intratracheal administration also showed significant antiviral and anti-inflammatory effects [39]. Therefore, due to the differences in the source of EVs and the administration schemes of EVs, the therapeutic effects for lung injuries caused by different etiologies may vary.

This study has several limitations: (1) While existing research indicates that day 7 post-infection represents the peak period for pulmonary inflammation and viral load [40], and multiple studies [41,42] have established this time point as a critical endpoint for evaluating antiviral and anti-inflammatory therapies, including inflammatory resolution, pathological changes, and treatment efficacy, the values measured at day 7 do not directly correlate with clinical outcomes such as survival rate or recovery status. Relevant therapeutic effects may only become apparent between days 14 and 21. Future studies should therefore include complete survival curves and body weight recovery data to enhance the comprehensiveness of the findings. (2) The current study used differential ultracentrifugation for the extraction of hUCMSC-EVs based on feasibility and accessibility. However, this method may introduce trace amounts of residual protein contaminants [43]. Subsequent work will employ more advanced separation techniques, such as size exclusion chromatography or density gradient ultracentrifugation, to improve the purity of hUCMSC-EVs. (3) Viral titers in the lungs were not assessed via 50% tissue culture infectious dose (TCID_50_) or real-time quantitative polymerase chain reaction (RT-qPCR). As a result, it was not possible to evaluate whether hUCMSC-EVs influence viral replication. Future studies will include quantitative viral load analyses to determine whether hUCMSC-EVs exert antiviral effects against influenza virus A (H1N1) infection. (4) The anti-inflammatory effects of hUCMSC-EVs were validated using only a single administration route in influenza virus A (H1N1)-infected mice. The absence of control groups, such as MSC-EVs derived from different sources or administered via different routes, limits the generalizability of the findings. Follow-up research will systematically compare EVs from MSCs of various origins and evaluate multiple administration routes to identify the optimal therapeutic strategy. (5) Only female mice were used in this study. This selection was based on previous reports indicating that female mice exhibit a stronger and more stable immune response to influenza infection, thereby improving the sensitivity for detecting treatment effects [44]. However, the exclusion of male mice may affect the broader applicability of the results. Subsequent studies will include male mice to evaluate the generalizability of the intervention. (6) Previous studies have demonstrated that Wharton’s jelly-derived MSC-EVs (WJ-EVs) can transfer miR-146a to recipient lung epithelial cells (EpiC), impairing TRAF6 and IRAK1 function. This leads to downregulation of the NF-κB pathway, reduced secretion of inflammatory cytokines, restoration of epithelial–endothelial crosstalk, and decreased immune cell recruitment [45], highlighting the important role of MSC-EVs cargo in mediating therapeutic effects. Future research will use multi-omics approaches to characterize the contents of hUCMSC-EVs and identify key molecular mediators of their therapeutic effects against influenza virus A (H1N1) infection. These aspects warrant further exploration in subsequent studies.

In conclusion, hUCMSC-EVs demonstrated a significant anti-inflammatory effect in mice infected with the influenza virus A (H1N1). This study provides a theoretical basis for the application of hUCMSC-EVs in lung inflammation and ALI caused by influenza virus A (H1N1) and offers more options for the prevention and treatment of influenza virus A (H1N1).

## 4. Materials and Methods

### 4.1. Experimental Reagents

MSC Basal Medium (Dakewe, Beijing, China, #6114011); Fetal bovine serum (FBS) for MSCs (XP BioMed, Shanghai, China, #C04400-500); exosome-depleted FBS (Umibio, Shanghai, China, #UR50202); Human mesenchymal stem cell adipogenic differentiation culture medium (Dakewe, Beijing, China, #6114531); Human mesenchymal stem cell osteogenic differentiation culture medium (Dakewe, Beijing, China, #6114541); oil red O staining solution (Dakewe, Beijing, China, #4060711); Alizarin red staining solution (Dakewe, Beijing, China, #4060611); 4% paraformaldehyde histiocytic fixative (Dingguo Changsheng, Beijing, China, #AR-0212); H&E Staining Kit (Beyotime, Shanghai, China, #C0105S); Ethanol Hydrochloride Ultra-Fast Differentiation Solution (Beyotime, Shanghai, China, #C0165M); Tris/EDTA Antigen Repairing Solution (ZSGB-BIO, Beijing, China, #ZLI-9069); TNF-α, IL-1β, IL-6 ELISA kit (ColorfulGene Biotech, Wuhan, China, #JYM0218Mo, #JYM0531Mo, #JYM0012Mo); primary antibodies: rabbit anti-CD9 (Abcam, Cambridge, UK, #ab263019), anti-CD63 (Abcam, Cambridge, UK, #ab134045), anti-TSG101 (Abcam, Cambridge, UK, #ab125011), anti-TNF-α (Abcam, Cambridge, UK, #ab307164), anti-IL-1β (Abcam, Cambridge, UK, #ab315084), anti-IL-6 (Abcam, Cambridge, UK, #ab290735); rabbit two-step assay kit (ZSGB-BIO, Beijing, China, #PV-9001); DAB color development kit (ZSGB-BIO, Beijing, China, #ZLI-9018).

### 4.2. Experimental Animals

Thirty female BALB/c mice (6 weeks old, body weight 18–20 g, specific pathogen free (SPF) grade) were purchased from Changchun Yisi Laboratory Animal Technology Co., Ltd., Changchun, China (License No. SYXK(Ji)2023-0010) and housed in the SPF-grade Animal Experiment Centre of the School of Basic Medical Sciences of Jilin University. All protocols involving animal experiments in this experiment were approved by the Ethics Committee for Animal Experiments of the College of Basic Medical Sciences of Jilin University (Approval No.2025-393).

### 4.3. Virus Strain

The H1N1 (FM1, A/FortMonmouth/1/47) strain was provided by the Department of Pathogen Biology, School of Basic Medical Sciences, Jilin University. The virus had haemagglutination potency of 1:256 to 1:512, mouse viral LD_50_ of 10^−5.5^, TCID_50_ of 10^−4.1^. Aliquots were stored at −80 °C until use.

### 4.4. Culture of hUCMSCs

hUCMSCs (Sciencell, Carlsbad, CA, USA, #7530) were maintained in MSC basal medium (Dakewe, Beijing, China, #6114011) supplemented with 10% FBS (XP BioMed, Shanghai, China, #C04400-500) (MSC complete culture medium) at 37 °C under 5% CO_2_. Cells were passaged at 80–90% confluence and used for experiments at passages 3–7.

### 4.5. Charaterization of hUCMSCs

Cell phenotype was confirmed by flow cytometry. Briefly, cells (1 × 10^6^/mL in PBS) were incubated with antibodies against CD34, CD45, CD73, CD90, and CD105 for 30 min at 4 °C (protected from light) After washing, cells were resuspended in 100 µL PBS and analyzed.

### 4.6. Multidirectional Differentiation of hUCMSCs

To induce adipogenic and osteogenic differentiation, 1 × 10^4^ hUCMSCs were inoculated per well and placed in 6-well plates coated with gelatin. Then, the MSC complete medium was added. When the cell density reached 80–90%, the cells were cultured in adipogenic differentiation induction medium (Dakewe, Beijing, China, #6114531) and osteogenic differentiation induction medium (Dakewe, Beijing, China, #6114541) for 18–21 days. The medium was replaced regularly during this period. After 18–21 days of differentiation induction, the cells were fixed with 4% paraformaldehyde and stained with oil red O (Dakewe, Beijing, China, #4060711) or Safranin O (Dakewe, Beijing, China, #4060611). Finally, images were captured using an inverted microscope (OLYMPUS IX71, Tokyo, Japan).

### 4.7. Isolation and Characterization of hUCMSC-EVs

The 3rd–7th generation hUCMSCs were inoculated at a density of 5 × 10^5^ cells per 10 cm culture dish, and MSC complete culture medium was added. The cells were cultured for 1–2 days until the cell fusion degree reached 60–70%. The upper layer medium was removed, and the cells were rinsed 2–3 times with phosphate-buffered saline (PBS) (Gibco, Paisley, Scotland, UK, #10010023), then 10 mL of exosome-depleted medium (MSC basal medium (Dakewe, Beijing, China, #6114011) supplemented with 10% exosome-depleted FBS (Umibio, Shanghai, China, #UR50202)) was added for 48 h to collect the conditioned medium. The conditioned medium was subjected to gradient centrifugation: 3000× *g* for 20 min (4 °C) → 10,000× *g* for 45 min (4 °C) → 0.22 µm filtration → ultracentrifugation at 120,000× *g* for 120 min (4 °C), and the supernatant was discarded. EVs resuspension: pellets were resuspended in 100 µL PBS, quantified by BCA assay, and stored at −80 °C. After gradient ultracentrifugation of the 180 mL of conditioned medium collected, approximately 720 μg of EVs could be obtained. This process was repeated to obtain a sufficient amount for administration. The morphology and size distribution of EVs were examined by TEM and nanoparticle tracking analysis (NTA). In addition, the representative EV markers CD9, CD63, and TSG101 were detected by Western blotting.

### 4.8. TEM Observation of hUCMSC-EVs Morphology

A total of 20–30 μL of hUCMSC-EVs suspension were uniformly dispensed onto a 200-mesh copper grid that had been pre-coated with a Formvar film. The grid was allowed to stand at room temperature for 3–5 min, after which excess liquid was blotted away with filter paper. Subsequently, 20 μL of 2% phosphotungstic acid solution (pH 7.0, filtered through a 0.22 μm membrane) was applied for negative staining, and the grid was left to stand for another 3–5 min. Excess staining solution was carefully blotted away with filter paper, and the grid was dried under an incandescent lamp. Once the sample was completely dried, the morphology of hUCMSC-EVs was imaged and photographed using a transmission electron microscope (Tecnai Spirit Biotwin, FEI, Eindhoven, The Netherlands) operated at 80 kV.

### 4.9. Western Blotting

Total protein was extracted from hUCMSCs and hUCMSC-EVs using RIPA lysis buffer containing phenylmethanesulfonyl fluoride (PMSF). The protein-containing lysate mixed thoroughly with 5-fold protein loading buffer and incubated at 37 °C for 30 min. In total, 20 μg of total protein from each sample were separated on a 12% sodium dodecyl sulfate (SDS) polyacrylamide gel and transferred to a PVDF membrane. The membrane was blocked with a protein-free quick-blocking solution (Servicebio, Wuhan, China, #G2052-500ml) and the primary antibodies were gently shaken and incubated overnight at 4 °C. The primary antibodies used included anti-CD9 antibody (1:1000, Abcam, Cambridge, UK), anti-TSG101 antibody (1:5000, Abcam, Cambridge, UK), anti-CD63 antibody (1:5000, Abcam, Cambridge, UK), and anti-Calnexin antibody (1:2500, Abcam, Cambridge, UK). After incubation, HRP-labeled goat anti-rabbit IgG (1:5000, Proteintech Group, Wuhan, China) was added and incubated at room temperature for 2 h. The protein bands were detected using enhanced ECL HRP substrate (Thermo Scientific, Waltham, MA, USA, #34580).

### 4.10. Influenza Virus A (H1N1) Infection Model and Treatment

Mice were randomly divided into NC group (no infection), H1N1-infected (model) group, and hUCMSC-EVs-treated group (n = 10/group) (Based on the preliminary experiment results, α = 0.05, test power (1 − β) = 85% calculation). Mice were anesthetized (1% sodium pentobarbital, 50 mg/kg) and inoculated intranasally with 50 µL H1N1 (10 LD50). Referring to the treatment procedures and dosages of other MSC-EVs [31], the hUCMSC-EVs-treated group received 70 µg EVs via tail vein at 24 h post-infection (BCA method measured EVs protein concentration as 0.35 mg/mL, with a dosing volume of 200 µL), followed by daily injections for 4 days. Body weights, temperatures, and clinical scores were recorded daily by different people for seven days to avoid subjective judgment and bias. Mice were anesthetized with 3 mL/kg sodium pentobarbital, and lungs/blood were collected. The lung index = (lung weight/body weight) × 100%.

Clinical scoring was performed using the following methods: (1) slightly ruffled fur; (2) ruffled fur and reduced mobility; (3) ruffled fur, reduced mobility, and rapid respiration; (4) ruffled fur, reduced mobility, hunched posture, and labored breathing and (5) death [31].

### 4.11. H&E Staining and Lung Pathological Scoring

The paraffin-embedded lung tissue sections were deparaffinized and rehydrated, and then stained with H&E. Three non-overlapping areas were randomly selected from each section, and scored by two experienced pathologists using a blind method under an optical microscope. The scoring criteria are as follows: 0, normal tissue; 1, mild inflammatory cell infiltration; 2, no significant destruction of lung structure; 3, thickening of alveolar septa; 4, severe pneumonia resulting in destruction of lung structure; 5, pneumonia covering the entire lung [42,46].

### 4.12. Enzyme-Linked Immunosorbent Assay (ELISA)

The mice serum was allowed to naturally coagulate at room temperature for 10–20 min and was then centrifuged at 3000× *g* for 20 min at 4 °C. The supernatant was collected for subsequent experiments. The ELISA kit (ColorfulGene Biotech, Wuhan, China) was used to measure the concentrations of TNF-α, IL-1β, and IL-6 in the serum, following the manufacturer’s instructions. First, 40 μL of sample diluent was added to the sample well, followed by 10 μL of the serum sample to be tested, resulting in a fivefold dilution of the sample. Each group comprised six independent serum samples, with triplicate determinations performed for each group. The BioTek Synergy H1 full-featured microplate reader (BioTek, Winooski, VT, USA) was used to measure the absorbance value of each well at a wavelength of 450 nm, and the final calculation was performed according to the operating instructions.

### 4.13. Immunohistochemical Staining

Immunohistochemical staining was performed to detect the expression of TNF-α, IL-1β, and IL-6 proteins in lung tissues. Paraffin sections were dewaxed and rehydrated, then immersed in distilled water. Antigen retrieval was performed using a microwave-heated Tris/EDTA buffer (pH 9.0). Sections were blocked with 10% goat serum at room temperature for 30 min. Incubate the tissue sections together with rabbit anti-TNF-α antibody (1:1000, Abcam, Cambridge, UK), rabbit anti-IL-1β antibody (1:1500, Abcam, Cambridge, UK), and rabbit anti-IL-6 antibody (1:100, Abcam, Cambridge, UK) at 4 °C overnight. Add an appropriate amount of reaction enhancer solution and incubate at 37 °C for 20 min. Add an appropriate amount of HRP-conjugated goat anti-rabbit IgG polymer and incubate at room temperature for 1 h. Treat the sections with freshly prepared DAB coloring solution at room temperature in the dark for 8 min, then counterstain the sections with hematoxylin. They were then photographed under a microscope, with three non-repetitive fields selected and photographed at high magnification. The appearance of brownish yellow in the cytoplasm or cytoplasm was considered positive, and the average expression area of the positive signal was analyzed by ImageJ (Version 1.8.0) software (NIH, Bethesda, MD, USA).

### 4.14. Statistical Analysis

Plotting with GraphPad 8.0 software. Data were expressed as mean values ± standard deviation. An unpaired two-tailed Student’s *t*-test was used for comparisons between two groups. For comparisons among multiple experimental groups, one-way ANOVA was used. In all statistical methods, a *p* value of <0.05 was considered statistically significant (* *p* value < 0.05, ** *p* value < 0.01, *** *p* value < 0.001, **** *p* value < 0.0001).

## Data Availability

The original contributions presented in this study are included in the article and Supplementary Materials. Further inquiries can be directed to the corresponding author.

## Supplementary Materials

The following supporting information can be downloaded at https://www.mdpi.com/article/10.3390/ijms26188839/s1.

## References

[1] Almutairi F., Sarr D., Tucker S.L., Fantone K., Lee J.K., Rada B. (2021). RGS10 Reduces Lethal Influenza Infection and Associated Lung Inflammation in Mice. Front. Immunol..

[2] Liu M., Zhao F., Xu J., Zhu X., Zhao Y., Wen R., Anirudhan V., Rong L., Tian J., Cui Q. (2023). Qingjin Huatan decoction protects mice against influenza a virus pneumonia via the chemokine signaling pathways. J. Ethnopharmacol..

[3] Choi A., García-Sastre A., Schotsaert M. (2020). Host immune response-inspired development of the influenza vaccine. Ann. Allergy Asthma Immunol..

[4] Tanner A.R., Dorey R.B., Brendish N.J., Clark T.W. (2021). Influenza vaccination: Protecting the most vulnerable. Eur. Respir. Rev..

[5] Moss R.B., Davey R.T., Steigbigel R.T., Fang F. (2010). Targeting pandemic influenza: A primer on influenza antivirals and drug resistance. J. Antimicrob. Chemother..

[6] Kayani M.D. (2022). Oseltamivir-resistant h1n1 influenza virus: Case report. J. Pak. Med. Assoc..

[7] Ding W., Li R., Song T., Yang Z., Xu D., Huang C., Shen S., Zhong N., Lai K., Deng Z. (2024). AMG487 alleviates influenza A (H1N1) virus-induced pulmonary inflammation through decreasing IFN-γ-producing lymphocytes and IFN-γ concentrations. Br. J. Pharmacol..

[8] Fu X., Liu G., Halim A., Ju Y., Luo Q., Song A.G. (2019). Mesenchymal Stem Cell Migration and Tissue Repair. Cells.

[9] Naji A., Eitoku M., Favier B., Deschaseaux F., Rouas-Freiss N., Suganuma N. (2019). Biological functions of mesenchymal stem cells and clinical implications. Cell. Mol. Life Sci..

[10] Harrell C.R., Jovicic N., Djonov V., Arsenijevic N., Volarevic V. (2019). Mesenchymal Stem Cell-Derived Exosomes and Other Extracellular Vesicles as New Remedies in the Therapy of Inflammatory Diseases. Cells.

[11] Arabpour M., Saghazadeh A., Rezaei N. (2021). Anti-inflammatory and M2 macrophage polarization-promoting effect of mesenchymal stem cell-derived exosomes. Int. Immunopharmacol..

[12] Wu R., Fan X., Wang Y., Shen M., Zheng Y., Zhao S., Yang L. (2022). Mesenchymal Stem Cell-Derived Extracellular Vesicles in Liver Immunity and Therapy. Front. Immunol..

[13] Nikfarjam S., Rezaie J., Zolbanin N.M., Jafari R. (2020). Mesenchymal stem cell derived-exosomes: A modern approach in translational medicine. J. Transl. Med..

[14] Dong L., Wang Y., Zheng T., Pu Y., Ma Y., Qi X., Zhang W., Xue F., Shan Z., Liu J. (2021). Hypoxic hUCMSC-derived extracellular vesicles attenuate allergic airway inflammation and airway remodeling in chronic asthma mice. Stem Cell Res. Ther..

[15] Dong B., Wang C., Zhang J., Zhang J., Gu Y., Guo X., Zuo X., Pan H., Hsu A.C., Wang G. (2021). Exosomes from human umbilical cord mesenchymal stem cells attenuate the inflammation of severe steroid-resistant asthma by reshaping macrophage polarization. Stem Cell Res. Ther..

[16] Abbaszadeh H., Ghorbani F., Abbaspour-Aghdam S., Kamrani A., Valizadeh H., Nadiri M., Sadeghi A., Shamsasenjan K., Jadidi-Niaragh F., Roshangar L. (2022). Chronic obstructive pulmonary disease and asthma: Mesenchymal stem cells and their extracellular vesicles as potential therapeutic tools. Stem Cell Res. Ther..

[17] Maremanda K.P., Sundar I.K., Rahman I. (2019). Protective role of mesenchymal stem cells and mesenchymal stem cell-derived exosomes in cigarette smoke-induced mitochondrial dysfunction in mice. Toxicol. Appl. Pharmacol..

[18] Zhou Y., Jin L., Lai X., Li Y., Sheng L., Xie G., Fang J. (2024). Adverse events associated with oseltamivir and baloxavir marboxil in against influenza virus therapy: A pharmacovigilance study using the FAERS database. PLoS ONE.

[19] Xu Y., Liu L. (2017). Curcumin alleviates macrophage activation and lung inflammation induced by influenza virus infection through inhibiting the NF-κB signaling pathway. Influenza Other Respir. Viruses.

[20] Li R., Han Q., Li X., Liu X., Jiao W. (2024). Natural Product-Derived Phytochemicals for Influenza A Virus (H1N1) Prevention and Treatment. Molecules.

[21] Guo C.Y., Wang Y., Feng Q., Sun L.J., Feng Y.M., Dong Y.H., Xu C.X. (2025). Umbilical Cord Mesenchymal Stem Cells Could Reduce Lung Damage Caused by H1N1 Influenza Virus Infection. J. Med. Virol..

[22] Tan Y., Wang Y., Souza-Moreira L., Wang C., Murray A.B.P., Salkhordeh M., Florian M., McIntyre L., Stewart D.J., Mei S.H.J. (2023). Mesenchymal stem cells induce dynamic immunomodulation of airway and systemic immune cells in vivo but do not improve survival for mice with H1N1 virus-induced acute lung injury. Front. Bioeng. Biotechnol..

[23] Liu H., Li C., Zhang X., Chen H., Zhang Q., Zeng Y., Zheng S., Zou J., Zhao Y., Zheng X. (2024). BMSC-Exosomes attenuate ALP dysfunction by restoring lysosomal function via the mTOR/TFEB Axis to reduce cerebral ischemia-reperfusion injury. Exp. Neurol..

[24] Sang H., Zhao R., Lai G., Deng Z., Zhuang W., Wu M., Wu J. (2023). Bone marrow mesenchymal stem cell-derived exosomes attenuate the maturation of dendritic cells and reduce the rejection of allogeneic transplantation. Adv. Clin. Exp. Med..

[25] Chen Y., Wang L., Liu M., Zhao J., Xu X., Wei D., Chen J. (2023). Mechanism of exosomes from adipose-derived mesenchymal stem cells on sepsis-induced acute lung injury by promoting TGF-β secretion in macrophages. Surgery.

[26] Chu M., Wang H., Bian L., Huang J., Wu D., Zhang R., Fei F., Chen Y., Xia J. (2022). Nebulization Therapy with Umbilical Cord Mesenchymal Stem Cell-Derived Exosomes for COVID-19 Pneumonia. Stem Cell Rev. Rep..

[27] Zhang Y., Wang P., Geng Z., Bao L., Gao S., Sun J., Liu X., Yang X., Zhao R., Li S. (2024). Geniposide attenuates influenza virus-induced pneumonia by regulating inflammatory cytokines production. Evidences to elucidate the followed pathway. Phytomedicine.

[28] Gao T., Liu J., Huang N., Zhou Y., Li C., Chen Y., Hong Z., Deng X., Liang X. (2024). Sangju Cold Granule exerts anti-viral and anti-inflammatory activities against influenza A virus in vitro and in vivo. J. Ethnopharmacol..

[29] Ling L.J., Lu Y., Zhang Y.Y., Zhu H.Y., Tu P., Li H., Chen D.F. (2020). Flavonoids from Houttuynia cordata attenuate H1N1-induced acute lung injury in mice via inhibition of influenza virus and Toll-like receptor signalling. Phytomedicine.

[30] Li R., Qu S., Qin M., Huang L., Huang Y., Du Y., Yu Z., Fan F., Sun J., Li Q. (2023). Immunomodulatory and antiviral effects of Lycium barbarum glycopeptide on influenza a virus infection. Microb. Pathog..

[31] Lee J.H., Jeon H., Lötvall J., Cho B.S. (2024). Therapeutic potential of mesenchymal stem cell-derived extracellular vesicles in SARS-CoV-2 and H1N1 influenza-induced acute lung injury. J. Extracell. Vesicles.

[32] Liu P., Yang S., Shao X., Li C., Wang Z., Dai H., Wang C. (2024). Mesenchymal Stem Cells-Derived Exosomes Alleviate Acute Lung Injury by Inhibiting Alveolar Macrophage Pyroptosis. Stem Cells Transl. Med..

[33] Mizuta Y., Akahoshi T., Guo J., Zhang S., Narahara S., Kawano T., Murata M., Tokuda K., Eto M., Hashizume M. (2020). Exosomes from adipose tissue-derived mesenchymal stem cells ameliorate histone-induced acute lung injury by activating the PI3K/Akt pathway in endothelial cells. Stem Cell Res. Ther..

[34] Deng H., Zhu L., Zhang Y., Zheng L., Hu S., Zhou W., Zhang T., Xu W., Chen Y., Zhou H. (2022). Differential Lung Protective Capacity of Exosomes Derived from Human Adipose Tissue, Bone Marrow, and Umbilical Cord Mesenchymal Stem Cells in Sepsis-Induced Acute Lung Injury. Oxid. Med. Cell. Longev..

[35] Nagamura-Inoue T., He H. (2014). Umbilical cord-derived mesenchymal stem cells: Their advantages and potential clinical utility. World J. Stem Cells.

[36] Meng M., Zhang W.W., Chen S.F., Wang D.R., Zhou C.H. (2024). Therapeutic utility of human umbilical cord-derived mesenchymal stem cells-based approaches in pulmonary diseases: Recent advancements and prospects. World J. Stem Cells.

[37] Fan C.G., Zhang Q.J., Zhou J.R. (2011). Therapeutic potentials of mesenchymal stem cells derived from human umbilical cord. Stem Cell Rev. Rep..

[38] Chen J., Liu S., Zou J., Wang Y., Ge H., Hui Y., Huang S., Li W., Na W., Huang X. (2025). Comparison of efficacy of exosomes derived from human umbilical cord blood mesenchymal stem cells in treating mouse acute lung injury via different routes. Front. Pediatr..

[39] Khatri M., Richardson L.A., Meulia T. (2018). Mesenchymal stem cell-derived extracellular vesicles attenuate influenza virus-induced acute lung injury in a pig model. Stem Cell Res. Ther..

[40] Buchweitz J.P., Harkema J.R., Kaminski N.E. (2007). Time-dependent airway epithelial and inflammatory cell responses induced by influenza virus A/PR/8/34 in C57BL/6 mice. Toxicol. Pathol..

[41] Zhou B., Wang L., Yang S., Liang Y., Zhang Y., Pan X., Li J. (2023). Rosmarinic acid treatment protects against lethal H1N1 virus-mediated inflammation and lung injury by promoting activation of the h-PGDS-PGD(2)-HO-1 signal axis. Chin. Med..

[42] Zhang Y., Li J., Qiu Z., Huang L., Yang S., Li J., Li K., Liang Y., Liu X., Chen Z. (2024). Insights into the mechanism of action of pterostilbene against influenza A virus-induced acute lung injury. Phytomedicine.

[43] Théry C., Witwer K.W., Aikawa E., Alcaraz M.J., Anderson J.D., Andriantsitohaina R., Antoniou A., Arab T., Archer F., Atkin-Smith G.K. (2018). Minimal information for studies of extracellular vesicles 2018 (MISEV2018): A position statement of the International Society for Extracellular Vesicles and update of the MISEV2014 guidelines. J. Extracell. Vesicles.

[44] Robinson D.P., Lorenzo M.E., Jian W., Klein S.L. (2011). Elevated 17β-estradiol protects females from influenza A virus pathogenesis by suppressing inflammatory responses. PLoS Pathog..

[45] Anggraeni N., Vuong C.K., Silvia P., Fukushige M., Yamashita T., Obata-Yasuoka M., Hamada H., Ohneda O. (2024). Mesenchymal stem cell-derived extracellular vesicles reduce inflammatory responses to SARS-CoV-2 and Influenza viral proteins via miR-146a/NF-κB pathway. Sci. Rep..

[46] Bao S., Zou Y., Wang B., Li Y., Zhu J., Luo Y., Li J. (2015). Ginsenoside Rg1 improves lipopolysaccharide-induced acute lung injury by inhibiting inflammatory responses and modulating infiltration of M2 macrophages. Int. Immunopharmacol..

