# Supplementary material for: Extracellular Vesicles Derived from Human Umbilical Cord Mesenchymal Stem Cells Alleviated the Inflammatory Response in Mice Infected with the Influenza Virus A (H1N1)
Source: Int J Mol Sci. 2025 Sep 11;26(18):8839. doi: 10.3390/ijms26188839 (PMC12470093; doi:10.3390/ijms26188839)

Figure S1. SDS-PAGE gel treated with Coomassie Brilliant Blue staining and silver staining. (A) SDS-PAGE gel treated with Coomassie Brilliant Blue staining. (B) SDS-PAGE gel treated with silver staining.

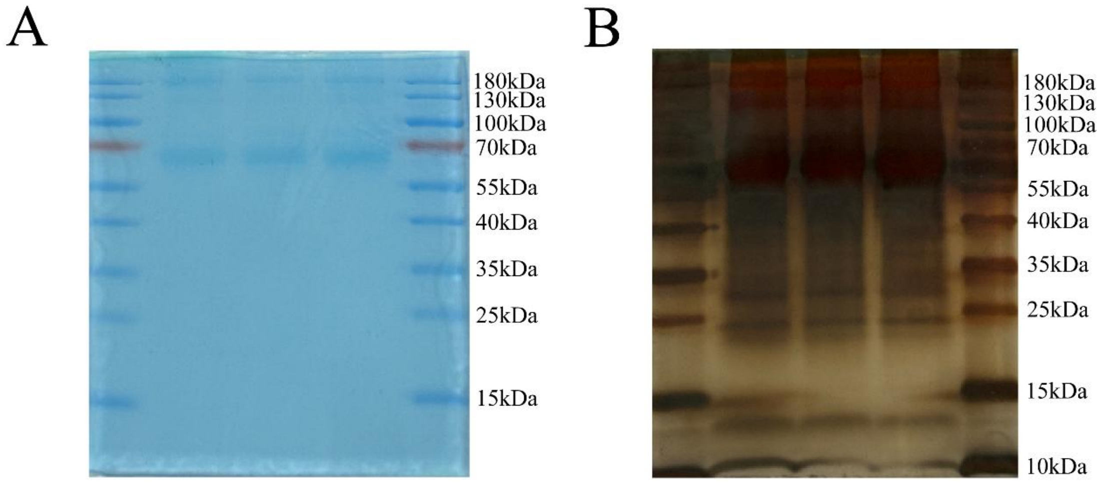

Supplement: Supplementary file 1 [file ijms-26-08839-s001.zip › ijms-3794293-supplementary.pdf]
